# PptAB Exports Rgg Quorum-Sensing Peptides in *Streptococcus*

**DOI:** 10.1371/journal.pone.0168461

**Published:** 2016-12-19

**Authors:** Jennifer C. Chang, Michael J. Federle

**Affiliations:** Department of Medicinal Chemistry and Pharmacognosy, Center for Biomolecular Sciences, College of Pharmacy, University of Illinois at Chicago, Chicago, Illinois, United States of America; University of Nevada School of Medicine, UNITED STATES

## Abstract

A transposon mutagenesis screen designed to identify mutants that were defective in peptide-pheromone signaling of the Rgg2/Rgg3 pathway in *Streptococcus pyogenes* generated insertions in sixteen loci displaying diminished reporter activity. Fourteen unique transposon insertions were mapped to *pptAB*, an ABC-type transporter recently described to export sex pheromones of *Enterococcus faecalis*. Consistent with an idea that PptAB exports signaling peptides, the pheromones known as SHPs (short hydrophobic peptides) were no longer detected in cell-free culture supernatants in a generated deletion mutant of *pptAB*. PptAB exporters are conserved among the *Firmicutes*, but their function and substrates remain unclear. Therefore, we tested a *pptAB* mutant generated in *Streptococcus mutans* and found that while secretion of heterologously expressed SHP peptides required PptAB, secretion of the *S*. *mutans* endogenous pheromone XIP (*sig**X* inducing peptide) was only partially disrupted, indicating that a secondary secretion pathway for XIP exists.

## Introduction

*Streptococcus pyogenes* (Group A streptococcus, GAS), is a human-restricted pathogen capable of causing both mild (pharyngitis, impetigo) and life-threatening (necrotizing fasciitis, sepsis) disease, but is also capable of an asymptomatic lifestyle in the host. All GAS genomes sequenced to date encode four Rgg-like transcription-factor proteins: Rgg1 (RopB), which controls the expression of the cysteine protease, SpeB [1,2,3]; Rgg2 and Rgg3, which together regulate genes that contribute to biofilm formation and lysozyme resistance [4,5]; and ComR, which occurs as one of two alleles (M1 or M3) and whose regulon includes genes associated with genetic competence [6]. Rgg proteins are cytoplasmic receptors for peptide pheromones, and with the exception of Rgg1, the pheromones that regulate the activity of each Rgg in GAS have been described [5,6]. Located next to and divergently transcribed from *rgg2* and *rgg3*, are two small ORFs, *shp2* and *shp3*, which encode 22 and 23 amino acid pro-peptide progenitors of the mature pheromones SHP2 and SHP3 (Table 1). In contrast, the small ORF *comS*, which encodes a 31 or 32 (depending on the allele) amino acid pro-peptide, lies directly downstream of its cognate regulator, *comR* (Table 1). The active pheromone derived from ComS is termed XIP, and in GAS comprises the C-terminal eight amino acids of the polypeptide.

**Table 1:** Peptide pheromones known or tested as substrates of PptAB.

Peptide	Source	Sequence of precursor (mature peptide)	Reference
cOB1	*E*. *faecalis*	MKKRTLWSVITVAVAVLVLGACGNKKS… (272 amino acids in total)	[7]
SHP_*gbs1555*_	*S*. *agalactiae*	MKKINKALLFTLIMDILIIVGG	[8]
SHP2	*S*. *pyogenes*	MKKVNKALLFTLIMDILIIVGG	This study
SHP3	*S*. *pyogenes*	MKKISKFLPILILAMDIIIIVGG	This study
ComS	*S*. *pyogenes*, M1 allele	MLKKYKYYFIFAALLSFKVVQELSAVDWWRL	This study
ComS	*S*. *mutans*	MFSILTSILMGLDWWSL	This study

*Streptococcus mutans*, an oral pathogen and the primary species associated with dental caries, also possesses four Rgg-like regulators. In *S*. *mutans*, ComR (SMU.61) is the best-characterized example and has provided an ideal model to study transcriptional control of the alternative sigma factor SigX, the master regulator of competence [9]. As in GAS and other species containing *comRS*, the *S*. *mutans comS* is encoded downstream of *comR*; the active pheromone comprises the C-terminus of the polypeptide (Table 1). Importantly, ComS alleles across streptococcal species display distinct sequences and pro-peptide lengths [10,11]. Moreover, XIP and SHP pheromone groups are considerably dissimilar from one another.

Although Rgg regulators respond to different pheromones, control different genes, and do not appear to cross-talk, the circuitry required for induction of Rgg2/3 and ComR signaling utilize analogous, and sometimes shared, cellular components. Pheromones are imported into the cell via the oligopeptide permease, Opp, where they interact with their cognate Rgg and initiate gene expression, including at promoters of the pheromone genes, leading to auto-feedback [5,6,9]. Pro-peptides must be exported from the cell and processed before they can function as active signals. The metalloprotease Eep is required for SHP pheromone production in *S*. *pyogenes* [5], but is not required to produce XIP in *S*. *mutans* [12], and additional processing steps may occur in the final maturation of either peptide [13]. However, the mechanism by which XIP and SHP peptides are exported from the cell remains poorly understood for the model systems of *S*. *pyogenes* and *S*. *mutans*. Unlike other systems regulating bacteriocin production (e.g., *comCDE* in *S*. *mutans*, *sil* in GAS), no dedicated transporter has been identified [14,15].

Recently, an ABC transporter called PptAB was identified as contributing to sex pheromone production in *Enterococcus faecalis* [7], and a separate study confirmed a role for PptAB in an Rgg-SHP signaling pathway in *Streptococcus agalactiae* [8]. Here, we report the identification of *pptAB* in a genetic screen and expand the PptAB substrate list to include GAS SHPs.

## Materials and Methods

### Bacterial strains

*S*. *pyogenes* and *S*. *mutans* were grown in Todd-Hewitt broth (BD) supplemented with 0.2% yeast extract (Amresco) or a chemically-defined medium (CDM; [5,16]) as indicated; broth cultures were grown at 37x° C without shaking, and agar plates were cultured at 37° C with 5% CO_2_. All cloning was done in *E*. *coli* strain BH10c [17] which was routinely cultured in Luria-Bertani broth (BD) at 30° C with agitation. All strains were stored at -80° C in 20% glycerol. Antibiotics were added at the following concentrations when appropriate–*S*. *pyogenes*: chloramphenicol (Cm), 3 μg mL^-1^; erythromycin (Erm), 0.5 μg mL^-1^; kanamycin (Km), 200 μg mL^-1^; spectinomycin (Spec), 200 μg mL^-1^; *S*. *mutans*: Cm 7.5 μg mL^-1^; Erm, 1.5 μg mL^-1^; Km, 750 μg mL^-1^; Spec, 500 μg mL^-1^; and *E*. *coli*: Erm, 500μg mL^-1^; Spec, 150μg mL^-1^.

### Construction of mutant strains and plasmids

To delete *pptAB* in GAS, a 4094 bp region encompassing the genes and surrounding up- and downstream regions was amplified by PCR using primers JC316/JC317 and cloned into pFED760. *pptAB* were subsequently deleted by inverse PCR using primers JC318/JC319, and the kanamycin resistance gene, *aphA3*, was amplified with primers JC320/JC321 and inserted into PacI sites to make pJC251-kan. This knockout plasmid was electroporated into wild-type NZ131 and the Δ*rgg3* mutant (JCC131), and a two-step temperature dependent selection process was used to identify the mutants of interest [18]. Deletion of *pptAB* in *S*. *mutans* was accomplished similarly using primers JC403/JC404 to amplify the genomic region from wild-type UA159 (pJC297), JC405/JC406 for inverse PCR, and JC320/JC407 for *aphA3* cassette amplification. *S*. *mutans* strains were transformed with linear PCR product amplified from the resulting plasmid, pJC298, in CDM with the addition of synthetic XIP [9]. To complement the deletion of *pptAB* in GAS, a 1989 bp PCR fragment was amplified from NZ131 DNA using primers JC322/JC323 and cloned into the multi-copy shuttle vector, pLZ12-Sp, to create p*pptAB* (pJC252). pP_*recA*_-*shp2* (pJC350) and pP_*recA*_-*shp3* (pJC352) were constructed by cloning 120 bp or 194 bp fragments containing NZ131 *shp2* (primers JC495/SHP2-C9-rev-BglII) or *shp3* (primers JC175/JC427), respectively, into pJC303, a pLZ12-Sp-based vector containing the *recA* promoter directly upstream of a multiple cloning site.

### Luciferase assays

Starter cultures of strains of interest were prepared by growing isolated colonies to mid-log phase in CDM (OD600 = 0.5 to 0.6; Spectronic 20+; Thermo), adding glycerol to 20% and freezing at -80° C in aliquots. On the experiment day, starters were thawed and diluted into fresh CDM to a starting OD600 of 0.01 (*S*. *pyogenes*) or 0.025 (*S*. *mutans*) and incubated at 37° C. For luciferase assays measuring endogenous reporter activity, the OD600 was measured and counts per second (CPS) of 50 μL were assessed by luminometer (Turner BioSystems) after exposure to the decyl aldehyde substrate (Acros); relative light units (RLU) were calculated by dividing CPS by OD600 at each time point. Alternatively, strains of interest were dispensed into a 96-well clear-bottom plate (Greiner), a 1% decyl aldehyde solution was added to interstitial spaces, and the plate was incubated at 37° C with continuous shaking in a microplate reader (Synergy 2, Biotek) with collection of OD600 and luminescence measurements every 20 minutes. For luciferase assays measuring reporter-inducing activity in supernatants, donor cultures were diluted into fresh CDM as described above and grown to an OD600 of 0.5 to 0.6. Donor cells were pelleted by centrifugation, and the supernatants were sterilized by the addition of antibiotics or by filtration. Appropriate luciferase reporter strains were also grown to mid- to late-log phase then diluted into the clarified supernatants to a final OD600 of 0.05, and OD600 and CPS were measured every 30 minutes until maximum RLU were achieved.

## Results and Discussion

### A transposon screen identifies an ABC transporter, PptAB

**Fig 1:**
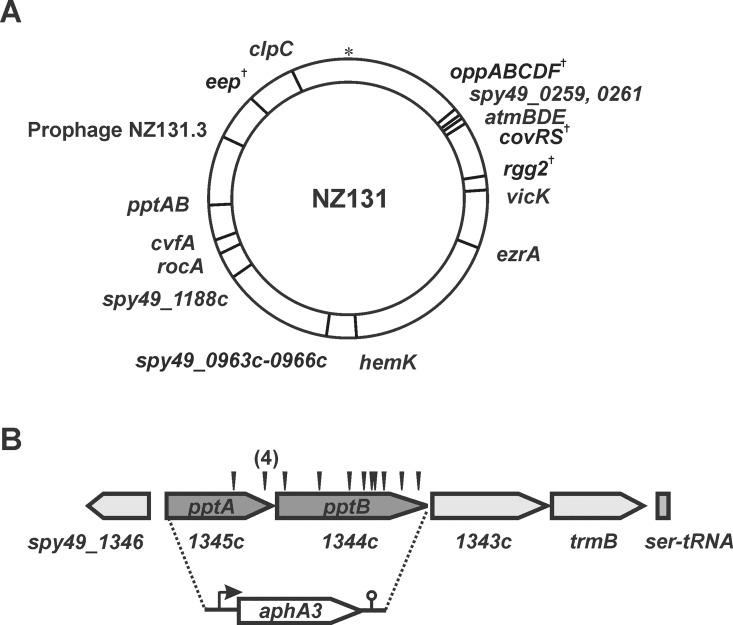
Transposon mutagenesis identifies novel components of the Rgg-SHP quorum sensing circuit. (A) Sixteen loci were identified according to our criteria (see text). Gene names and chromosomal location are indicated, with the replication origin (*) at twelve o’clock. Components of the QS circuit previously identified are indicated (^†^). (B) Fourteen unique insertions (arrowheads) mapped to *pptAB*, a predicted ABC transporter. *pptAB* were replaced with a cassette containing *aphA3*, which confers resistance to kanamycin.

We have previously shown that the primary targets of Rgg2/3-SHP regulation are the *shp* genes themselves along with their neighboring downstream genes [5]. To identify novel components required for Rgg-SHP signaling, we developed a genetic screen to identify genes involved in secretion, maturation, detection and degradation of SHP pheromones [19]. Briefly, a *mariner* transposon system [20] was used to mutagenize Δ*rgg3* strains containing luciferase (*lux*; JCC198) or β-glucuronidase (*gus*; JCC233) reporter genes downstream of *shp3*; in this background, P_*shp3*_ is highly expressed due to the absence of Rgg3, which normally acts as a repressor when pheromone levels are low. Approximately 10,000 individual mutants were screened for loss of reporter activity, and 16 loci were identified in which there were at least three independent transposon insertions from two or more rounds of screening, including genes previously found to be important for Rgg-SHP signaling (e.g., *rgg2*, *eep*, *opp*) [5] (Fig 1A). We recently described how *covRS*, one of the loci with the highest number of insertions (12 unique insertions), affects signaling through the regulation of the PepO protease [19]. Here, we report that a locus with 14 unique transposon insertions encodes the ABC transporter, PptAB (Fig 1B), an ABC transporter that was recently identified as the exporter for *Enterococcus faecalis* sex pheromones [7] and confirmed to function in the same capacity for *Streptococcus agalactiae* Rgg/SHP pheromones [8].

### PptAB is the primary transporter for SHP pheromones in GAS

**Fig 2:**
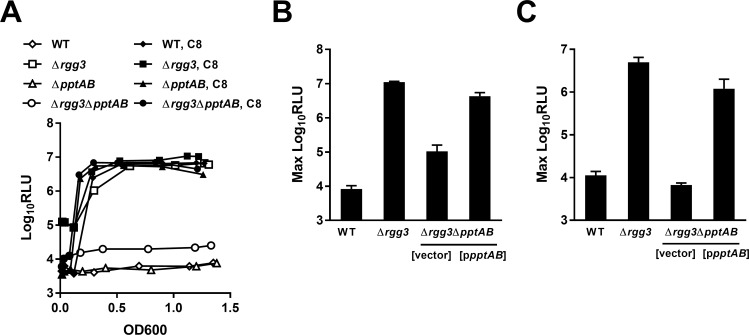
Rgg-SHP signaling in a *S*. *pyogenes pptAB* mutant. (A) Luciferase expression from P_*shp3*_ reporters integrated into wild-type (WT; NZ131), Δ*rgg3* (JCC131), and Δ*rgg3*Δ*pptAB* (JCC209) GAS strains with (closed symbols) and without (open symbols) the addition of 100nM synthetic SHP3-C8 (C8) peptide. Data shown are representative of experiments performed at least three times. (B) Maximum P_*shp3*_-*lux* reporter activity in WT, Δ*rgg3*, or Δ*rgg3*Δ*pptAB* strains carrying a plasmid encoding *pptAB* (pJC252) or empty vector (pLZ12-Sp). (C) Maximum P_*shp3*_-*lux* reporter-inducing activity in conditioned supernatants prepared from WT, Δ*rgg3*, or Δ*rgg3*Δ*pptAB* donor strains expressing *pptAB* (pJC252) or empty vector (pLZ12-Sp). Donor cultures were grown to OD ~0.5, cells were removed by centrifugation and filtration, and P_*shp3*_*-lux* activity of a Δ*rgg3 shp*_*GGG*_ reporter strain (BNL204) was measured. For B and C, data shown are the mean and SD from at least three experiments.

To confirm the role of *pptAB* in Rgg-SHP signaling in GAS, deletion mutants in which both genes were replaced with a kanamycin-resistance cassette were constructed in both the wild-type NZ131 and Δ*rgg3* backgrounds, and a P_*shp3*_-*lux* reporter (pJC219) was integrated into the chromosomes of the resulting strains. As expected, deletion of *pptAB* in the Δ*rgg3* background led to a >300-fold decrease in luciferase activity, although the Δ*rgg3*Δ*pptAB* mutant (JCC209) still had residual reporter activity ~3-fold higher than unstimulated wild-type and the single Δ*pptAB* mutant (JCC208) (Fig 2A). This residual activity is attributable to loss of direct repression by Rgg3 of the *shp* promoter, as has been documented in strains incapable of pheromone production [21]. Furthermore, *pptAB* mutants were still capable of robust P_*shp3*_-*lux* induction following the addition of synthetic SHP-C8 pheromone, confirming a role for *pptAB* in signal production but not signal detection. Interestingly, although *pptAB* are predicted to be co-transcribed as part of a multi-cistronic operon [22], no insertions were identified in downstream genes (*spy49_1343c* and *trmB*, a predicted thiamine kinase and tRNA (guanine-N(7)-)-methyltransferase, respectively), suggesting these genes are transcribed independently from a different promoter and/or they are dispensable for pheromone production. Additionally, the *pptAB* deletion in the Δ*rgg3* background could be complemented with *pptAB* alone (p*pptAB*; pJC252), further supporting the lack of a role for *spy49_1343* and *trmB* in Rgg-SHP signaling (Fig 2B). As SHP pheromones are secreted into the extracellular environment and can be detected in cell-free spent media from producer strains [5], the importance of PptAB for this process was confirmed by quantifying P_*shp3*_-*lux*-inducing activity in supernatants conditioned by wild-type, Δ*rgg3*, Δ*rgg3*Δ*pptAB* or complemented strains (Fig 2C).

**Fig 3:**
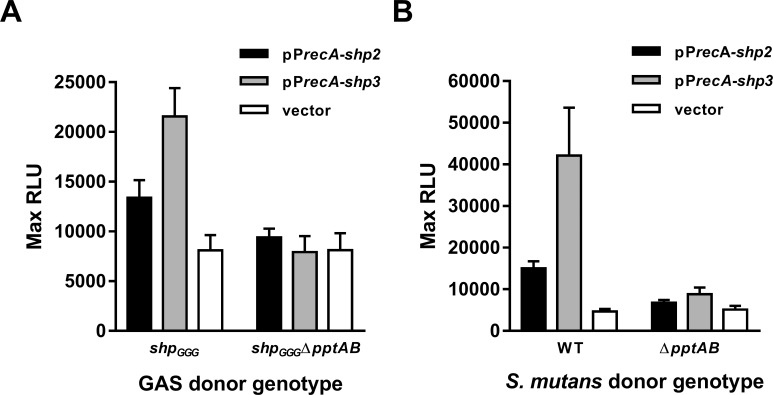
PptAB exports SHP pheromones in GAS and *S*. *mutans*. (A) Maximum P_*shp3*_-*lux* reporter-inducing activity in culture supernatants prepared from (A) GAS and (B) *S*. *mutans* WT and Δ*pptAB* donor strains expressing P_*recA*_-*shp2* (pJC350) or P_*recA*_-*shp3* (pJC352). Both GAS donor strains are deleted for chromosomal copies of *shp2* and *shp3* (*shp*_*GGG*_; BNL170 and JCC218, respectively). Donor cultures were grown to OD ~0.5, cells were removed by centrifugation, supernatants were filtered or chemically sterilized, and P_*shp3*_*-lux* activity of a Δ*rgg3 shp*_*GGG*_ reporter strain (BNL204) was measured. Data shown are the mean and SD from at least three experiments.

In GAS, transcription of *shp* genes is subject to auto-feedback, wherein induction of Rgg-SHP signaling leads to increased expression of the pheromones themselves [5]. To uncouple SHP production from the influence of Rgg2 and Rgg3 and to separate production of the pheromones from their downstream function, full-length *shp2* and *shp3* were cloned under the *recA* promoter (pP_*recA*_-*shp2*, pJC350; pP_*recA*_-*shp3*, pJC352), which is constitutively expressed. These plasmids were used to transform strains in which the start codons of both *shp* genes had been mutated to GGG (*shp*_*GGG*_; BNL170), rendering the strains unable to produce pheromone from chromosomal loci or undergo auto-induction. Spent culture supernatants were prepared from donor strains BNL170 or a *shp*_*GGG*_Δ*pptAB* strain (JCC218) carrying pJC350, pJC352, or the empty vector, and pheromone production was quantified by measuring the luciferase activity induced in a P_*shp3*_-*lux* reporter strain (BNL204). As expected, supernatants from *shp*_*GGG*_ but otherwise wild-type strains contained P_*shp3*_-inducing activity, but media from *shp*_*GGG*_ Δ*pptAB* or donors with empty vector did not (Fig 3A).

Interestingly, although both *shp2* and *shp3* were expressed from the same promoter (P_*recA*_), luciferase-inducing activity was always higher in pP_*recA*_-*shp3* supernatants. Our lab has previously shown that *shp3* has greater potential to activate Rgg-SHP signaling than *shp2*; using a series of gene replacements and by constructing chimeric *shp*s, it was determined that the difference in activation potential lies in the non-effector N-terminal portion of the peptides [23]. Furthermore, mature pheromones (C-terminal eight amino acids; Table 1) bind the Rgg proteins and induce P_*shp*_ signaling with similar EC_50_ values [13,23]. However, we have also shown that an aminopeptidase, PepO, degrades SHPs and limits signaling, with SHP2-C8 inactivated more efficiently than SHP3-C8 [19]. Thus, it is possible that the difference between SHP2- and SHP3-containing supernatants observed here arises from differences in processing or degradation, or even in efficiency of export by PptAB. However, there remains a significant reduction in activity in supernatants prepared from the Δ*pptAB* mutant versus wild-type (p<0.0001, Student’s t-test), regardless of SHP identity, confirming its importance in export of both SHP pheromones. Additionally, induction of signaling in wild-type (*shp*-intact) cells leads to positive feedback at both *shp* promoters; therefore, the functional consequences of differences between the two peptides are most likely minimal in wild-type cells under normal conditions.

### *S*. *mutans* PptAB exports GAS SHP pheromones

*pptAB* are conserved among *Firmicutes* within the orders *Bacillales* and *Lactobacillales*, including in several pathogenic species, and the transporter was previously shown to export signaling peptides in *E*. *faecalis* [7] and *S*. *agalactiae* [8] in addition to *S*. *pyogenes* as described above. PptA and PptB of GAS share 88% and 69% similarity with homologues in *S*. *mutans*. To test whether the *S*. *mutans* transporter could export heterologous SHP pheromones, wild-type (UA159) and Δ*pptAB* (JCC263) strains were transformed with the P_*recA*_-*shp2* and P_*recA*_-*shp3* expression constructs (pJC350 and pJC352, respectively), and the resulting strains were used to produce conditioned media. Similar to experiments with GAS donor strains, supernatants from wild-type cells contained higher levels of P_*shp3*_ reporter-inducing activity than those from Δ*pptAB* (Fig 3B), suggesting that the criteria by which this ABC transporter recognizes substrates are conserved across different species. Although the *S*. *mutans* genome encodes four Rgg-like regulators, with the exception of ComRS, the peptides and functions of these proteins have not been well characterized. However, given our findings, PptAB seems a possible candidate for transport of these substrates. As with GAS-conditioned supernatants, we observed greater luciferase-inducing activity in pP_*recA*_-*shp3* supernatants than pP_*recA*_-*shp2* supernatants. It is worth noting that both Eep, a metalloprotease involved in SHP processing [5], and PepO are conserved in *S*. *mutans*; therefore, the same factors that contribute to the differences between SHP2 and SHP3 observed in GAS may be in play in this species. Finally, although there was a significant reduction in activity from supernatants produced by wild-type versus Δ*pptAB* donor strains, supernatants from Δ*pptAB* still contained activity that was significantly different from the vector-only control (pP_*recA*_-*shp2*, p = 0.0002; pP*recA*-*shp3*, p<0.0001; Student’s t-test). Whether this difference is due to export, albeit inefficient, by another unidentified ABC transporter or to non-specific cell lysis during growth has not been determined.

### PptAB contributes to but is not required for export of competence pheromones

As mentioned above, both GAS and *S*. *mutans* possess homologues of ComR, an important regulator of competence [6,9]. These proteins are 68% similar and are activated upon binding their cognate XIP pheromone (Table 1). ComR activation induces expression of *sigX*, which is in turn required for expression of competence-related genes. Like the GAS *shp* promoter, robust *S*. *mutans* P_*sigX*_ induction depends on auto feedback of the ComRS sensory system, since in addition to P_*sigX*_, ComR positively regulates expression of *comS*. The importance of ComRS in the natural transformation of *S*. *mutans* has been demonstrated [9,24], and our lab has also shown that ComRS signaling is functional in GAS, as evidenced by the induction of a P_*sigX*_-*lux* reporter upon addition of synthetic XIP, although laboratory conditions favoring spontaneous P_*sigX*_ induction and transformation have remained elusive [6]. Recently, natural transformation was demonstrated for GAS grown in a biofilm model, suggesting that other host-derived factors/signals are required and our current *in vitro* conditions do not recapitulate the correct environment [25].

**Fig 4:**
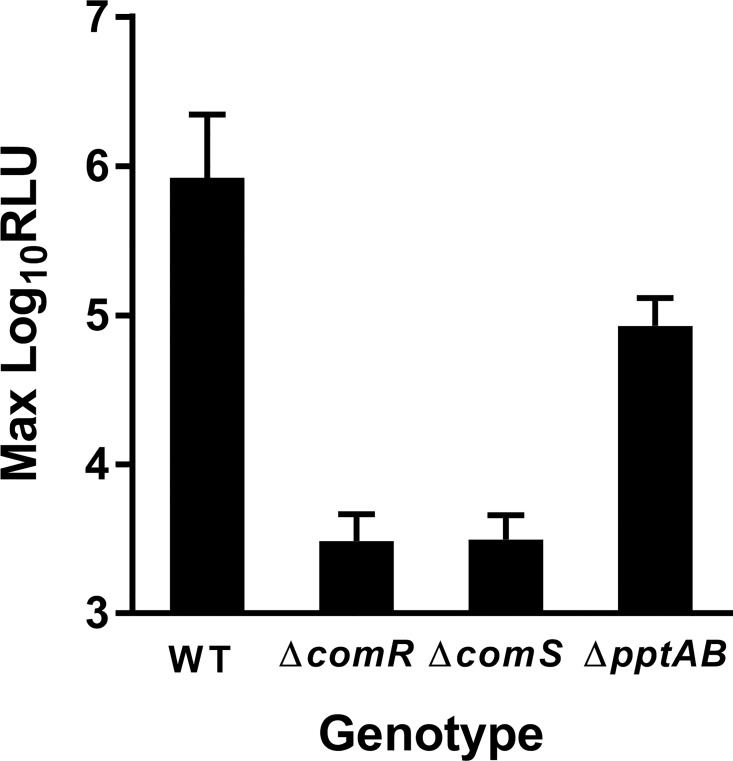
ComRS signaling in a *S*. *mutans pptAB* mutant. Maximum luciferase activity of WT (UA159), Δ*comR* (MW02), Δ*comS* (MW05) and Δ*pptAB* (JCC263) mutants carrying a multi-copy P_*sigX*_-*lux* reporter (pWAR304). Cells were grown to mid-log phase in CDM then diluted to an OD600 ~0.05 in a 96-well plate, and growth and luciferase activity were measured every 20 minutes in a Synergy 2 plate reader (Biotek). Data shown are the mean and SD from three experiments.

To ask whether PptAB is important for ComRS signaling, we took advantage of the observation that when grown in CDM, *S*. *mutans* spontaneously develops a high level of competence during late-logarithmic growth, an event that is slightly preceded by expression from the P_*sigX*_ promoter and coincides with the accumulation of XIP in conditioned supernatants [9,12,24,26]. The growth and luciferase activity of wild-type *S*. *mutans* and the *pptAB* mutant (UA159 and JCC263, respectively) containing a P_*sigX*_-*luxAB* reporter (pWAR304) were measured over time. The *pptAB* mutant exhibited an ~10-fold reduction in average maximum RLU compared to wild-type, but still had P_*sigX*_ activity ~25-fold greater than *comR* or *comS* mutants, which are defective for signaling suggesting that PptAB may contribute to, but is not required for, the production of XIP (Fig 4).

In an attempt to uncouple *comS* expression from the positive-feedback loop and to further explore the role of PptAB in ComS/XIP secretion, the GAS M1 allele, *comS*_M1_GAS_, or the *S*. *mutans* gene, *comS*_UA159_, was cloned downstream of the P_*recA*_ promoter to make pJC354 and pJC371, respectively. These plasmids were then transferred to donor strains. In NZ131, the native *comR* contains a duplication of three amino acids in the C-terminal domain of the protein rendering it nonfunctional. Therefore, a strain was constructed in which the NZ131 gene was replaced with a functional copy from MGAS8232 (called NZ131 *comR*_MGAS8232_ or MW361; see S1 File), and the ability of this strain to induce transcription from P_*sigX*_ in response to XIP was confirmed (S1 Fig). Unfortunately, conditioned supernatants collected from donor strains expressing P_*recA*_-*comS* varied widely in P_*sigX*_-inducing activity and failed to clarify the role of PptAB in ComS export (S2 and S3 Figs). For GAS ComS/XIP, conditions that lead to expression of the gene in vitro remain unknown, and endogenously-produced pheromone has never been detected. Thus, it is possible that even though transcription was under the control of a constitutive promoter, additional factors needed for productive pheromone synthesis are lacking in both GAS and *S*. *mutans* during growth under these conditions. For *S*. *mutans* ComS/XIP, supernatants collected at time points early enough to avoid P_*sigX*_ auto-induction may not have had time to accumulate detectable XIP in the supernatants. Furthermore, growth phase and culture pH can have a significant effect on cells’ response to ComS, thus it is possible the conditions tested here were not favorable for robust pheromone detection or production [27]. Finally, it has been proposed that processed XIPs in some streptococci remain in close association with the cell surface, which would complicate its detection by the method used here [28].

## Concluding Remarks

We have shown here that the ABC transporter, PptAB, plays an important role in the export of SHP pheromones in GAS, adding this species to a growing list organisms whose cell-cell signaling circuitry relies on this transporter for efficient export of signaling molecules from the cell. PptAB is conserved among many bacteria, including many *Firmicute* species. Indeed, we found that the *S*. *mutans* homologue could export GAS pheromones. Interestingly however, PptAB was not critical for ComRS signaling in this species, suggesting that the exporter must exhibit some substrate specificity and/or the competence pheromone preferentially uses an as-of-yet unidentified exporter. The *S*. *mutans* genome contains three Rgg-like regulators in addition to ComR, and the role of PptAB in the export of this species’ endogenous SHP pheromones remains to be determined. Finally, if PptAB has evolved to export signaling peptides, it is possible that some of the phenotypes exhibited by *pptAB* mutants in other species, including exoprotein secretion, competence, sporulation in *B*. *subtilis* [29,30,31], and cell wall structure and composition in *S*. *aureus* [32], may be regulated by small peptides.

## Supporting Information

S1 Fig NZ131 *comR*_MGAS8232_ responds to synthetic peptide in a dose-dependent manner. NZ131 in which the native *comR* allele was replaced with MGAS8232 *comR* (MW361) and carrying a multi-copy P_*sigX*_-*lux* reporter (pWAR200) was grown in CDM containing synthetic M1 GAS XIP at the indicated concentrations. OD600 and CPS were measured until maximum RLU were achieved.
(TIF)

S2 Fig P_*sigX*_-inducing activity in conditioned supernatants from strains expressing GAS *comS*. Maximum P_*sigX*_-*lux* reporter activity induced by supernatants from NZ131 *comR*_MGAS8232_ (MW361), UA159, and UA159Δ*pptAB* (JCC263) donor strains expressing the GAS M1 *comS* allele from the *recA* promoter (pJC354). Donor strains were grown to an OD600 of 0.5 to 0.6, and supernatants were clarified by centrifugation and the addition of erythromycin. NZ131 *comR*_MGAS8232_ containing the GAS P_*sigX*_-*lux* reporter (pWAR200) was diluted into the supernatants, and OD600 and CPS were measured until maximum RLU were achieved.
(TIF)

S3 Fig P_*sigX*_-inducing activity in conditioned supernatants from strains expressing UA159 *comS*. Maximum P_*sigX*_-*lux* reporter activity induced by conditioned supernatants from UA159 and UA159Δ*pptAB* (JCC263) donors expressing the *S*. *mutans comS* from the *recA* promoter (pJC371). Donor strains were grown to an OD600 of 0.5 to 0.6, and supernatants were clarified by centrifugation and the addition of erythromycin. A Δ*comS* strain containing the *S*. *mutans* P_*sigX*_-*lux* reporter (MW17) was diluted into the supernatants, and OD600 and CPS were measured until maximum RLU were achieved.
(TIF)

S1 File Supplementary Methods and References. Methods describing construction of strains and plasmids used for experiments in Supporting Information, and reference list for Supporting Information.
(DOCX)

S1 Table Strains and plasmids used in this study (DOCX)

S2 Table Primers used in this study (DOCX)
