# Supplementary figures and images for: PptAB Exports Rgg Quorum-Sensing Peptides in Streptococcus
Source: PLoS One. 2016 Dec 19;11(12):e0168461. doi: 10.1371/journal.pone.0168461 (PMC5167397; doi:10.1371/journal.pone.0168461)

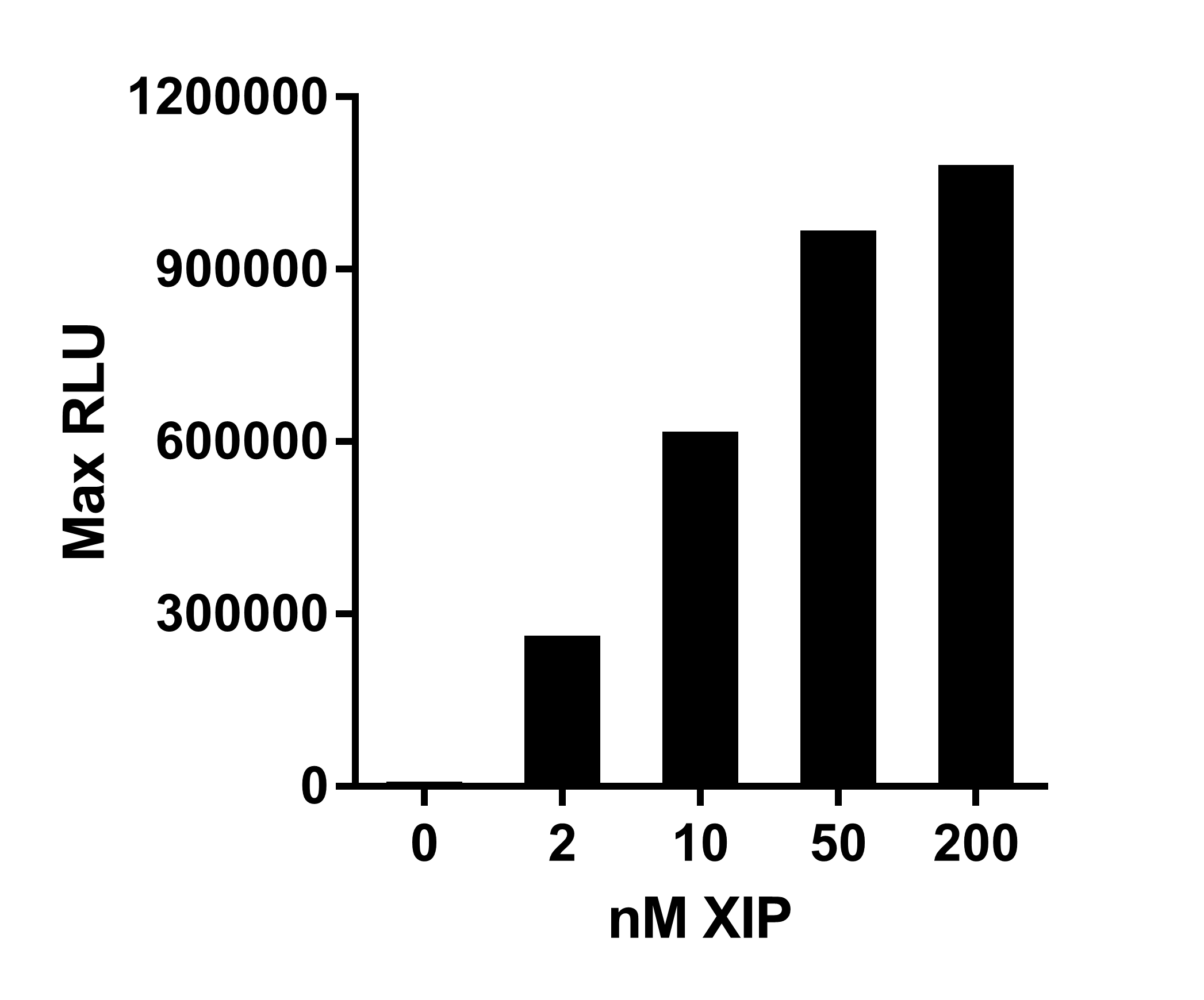

Supplement: S1 Fig — NZ131 in which the native comR allele was replaced with MGAS8232 comR (MW361) and carrying a multi-copy PsigX-lux reporter (pWAR200) was grown in CDM containing synthetic M1 GAS XIP at the indicated concentrations. OD600 and CPS were measured until maximum RLU were achieved. (TIF) [file pone.0168461.s001.tif]

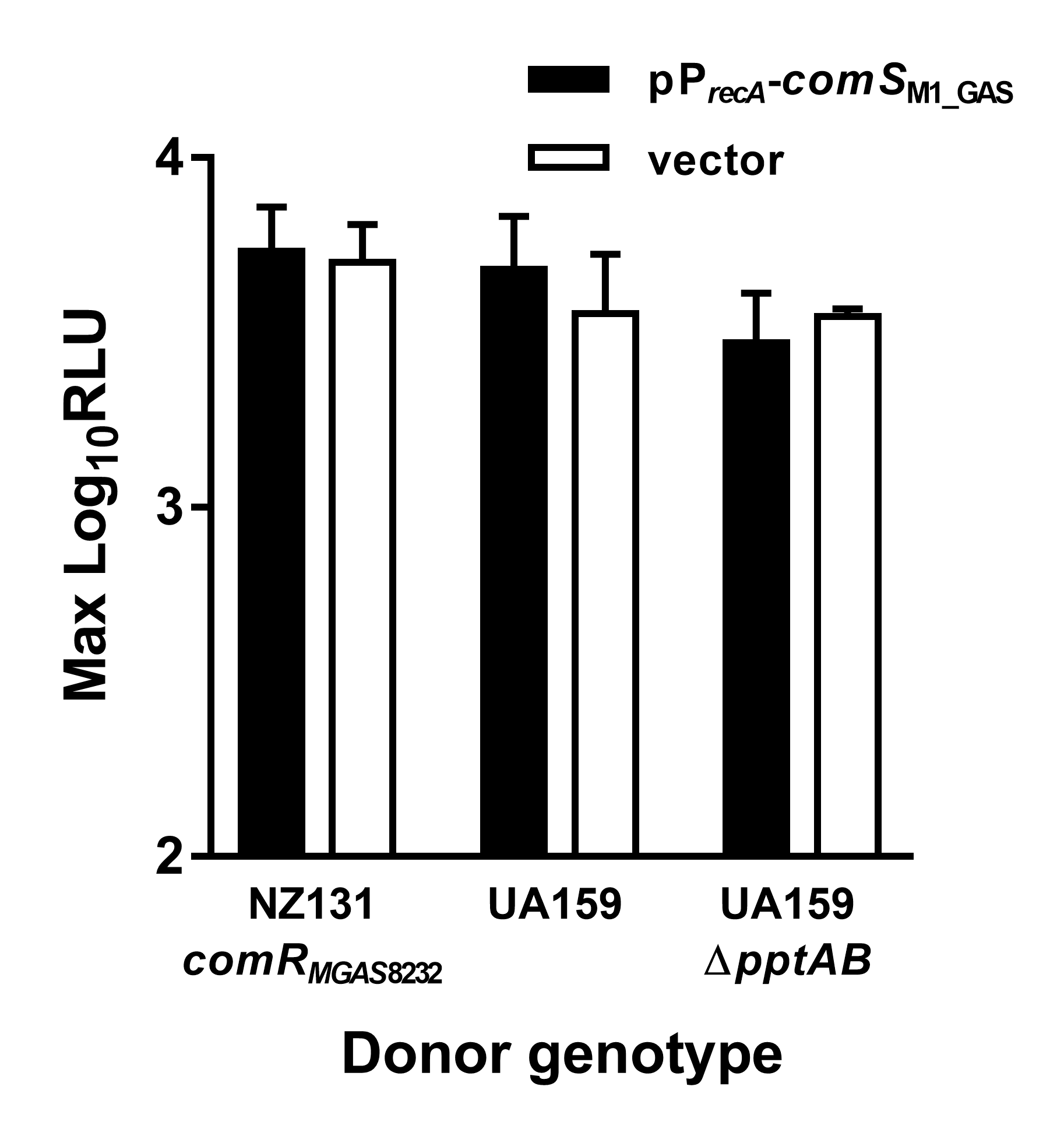

Supplement: S2 Fig — Maximum PsigX-lux reporter activity induced by supernatants from NZ131 comRMGAS8232 (MW361), UA159, and UA159ΔpptAB (JCC263) donor strains expressing the GAS M1 comS allele from the recA promoter (pJC354). Donor strains were grown to an OD600 of 0.5 to 0.6, and supernatants were clarified by centrifugation and the addition of erythromycin. NZ131 comRMGAS8232 containing the GAS PsigX-lux reporter (pWAR200) was diluted into the supernatants, and OD600 and CPS were measured until maximum RLU were achieved. (TIF) [file pone.0168461.s002.tif]

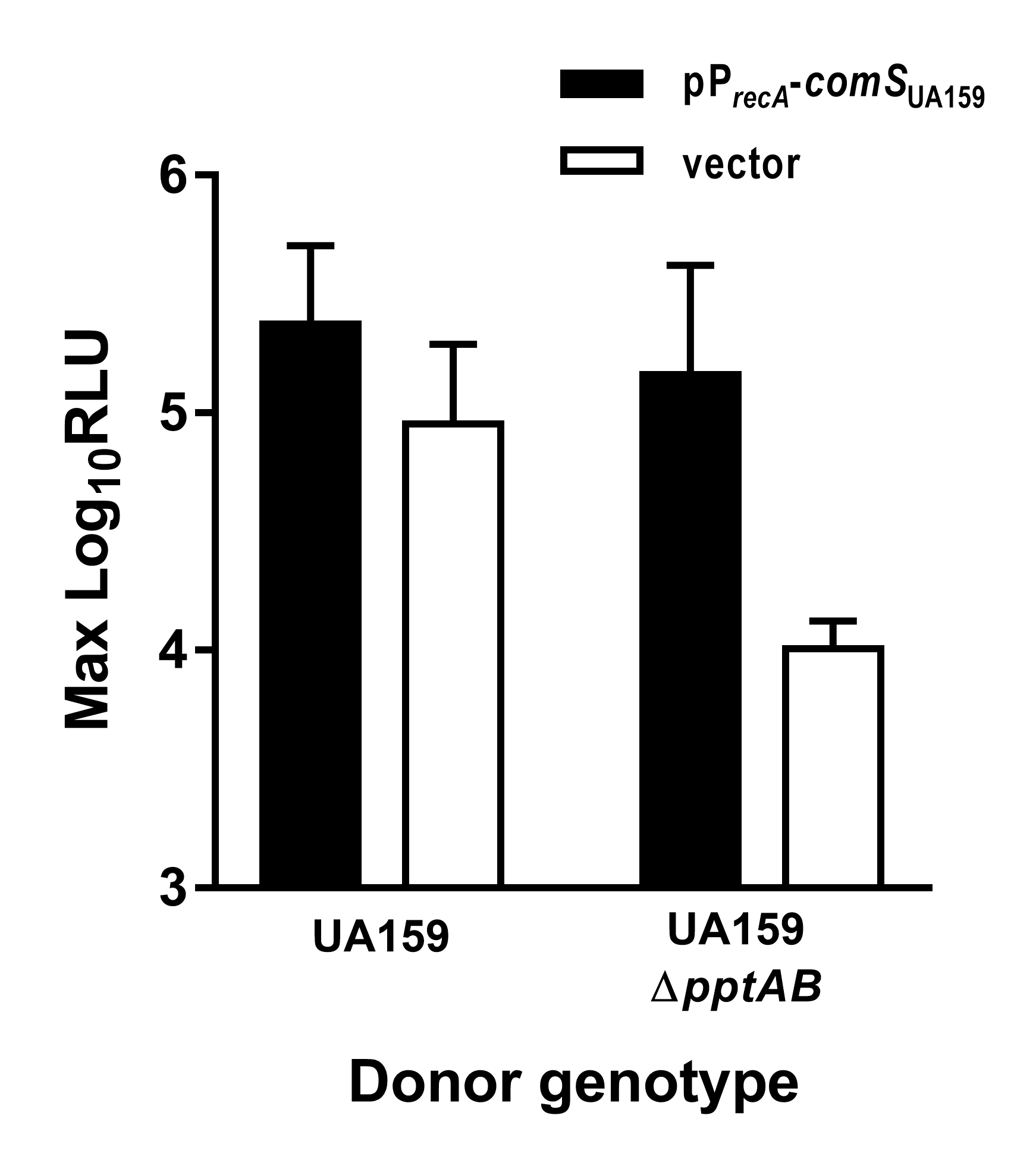

Supplement: S3 Fig — Maximum PsigX-lux reporter activity induced by conditioned supernatants from UA159 and UA159ΔpptAB (JCC263) donors expressing the S. mutans comS from the recA promoter (pJC371). Donor strains were grown to an OD600 of 0.5 to 0.6, and supernatants were clarified by centrifugation and the addition of erythromycin. A ΔcomS strain containing the S. mutans PsigX-lux reporter (MW17) was diluted into the supernatants, and OD600 and CPS were measured until maximum RLU were achieved. (TIF) [file pone.0168461.s003.tif]
