# Supplementary material for: PptAB Exports Rgg Quorum-Sensing Peptides in Streptococcus
Source: PLoS One. 2016 Dec 19;11(12):e0168461. doi: 10.1371/journal.pone.0168461 (PMC5167397; doi:10.1371/journal.pone.0168461)
Supplement: S1 File — Methods describing construction of strains and plasmids used for experiments in Supporting Information, and reference list for Supporting Information. (DOCX) [file pone.0168461.s004.docx]

**Supplemental Methods**

*Construction of strains and plasmids*

To replace the NZ131 *comR* allele which contains a duplication of three amino acids in the C-terminal domain of the protein, a NZ131 Δ*comR*::*cat* strain was first constructed using pWAR195 and the same two-step temperature dependent process described in the Methods. The *comR* gene from MGAS8232 was subsequently amplified using primers LMW30/LMW33 and cloned into pFED760. The resulting plasmid, pWAR289, was used to transform NZ131 Δ*comR*::*cat* and the two-step temperature dependent process was used again with screening for clones that became chloramphenicol sensitive and gained the corrected *comR*_MGAS8232_ gene as determined by sequencing; this strain was renamed MW361.  ComS overexpression plasmids pP*_recA_*-*comS*_M1_GAS_ (pJC354; primers JC426/498) and pP*_recA_*-*comS*_UA159_ (pJC371; primers JC511/512) were constructed by amplifying PCR fragments from NZ131 or UA159 DNA using the indicated primers and cloning these fragments into pJC303.

**References for Supporting Information**

1. Cook LC, LaSarre B, Federle MJ. Interspecies communication among commensal and pathogenic streptococci. MBio. 2013;4(4).

2. LaSarre B, Chang JC, Federle MJ. Redundant group a streptococcus signaling peptides exhibit unique activation potentials. J Bacteriol. 2013 Sep;195(18):4310-8.

3. Chang JC, LaSarre B, Jimenez JC, Aggarwal C, Federle MJ. Two group A streptococcal peptide pheromones act through opposing Rgg regulators to control biofilm development. PLoS Pathog. 2011 Aug;7(8):e1002190.

4. Wilkening RV, Chang JC, Federle MJ. PepO, a CovRS-controlled endopeptidase, disrupts Streptococcus pyogenes quorum sensing. Mol Microbiol. 2016 Jan;99(1):71-87.

5. Smoot JC, Barbian KD, Van Gompel JJ, Smoot LM, Chaussee MS, Sylva GL, et al. Genome sequence and comparative microarray analysis of serotype M18 group A Streptococcus strains associated with acute rheumatic fever outbreaks. Proc Natl Acad Sci U S A. 2002 Apr 2;99(7):4668-73.

6. McShan WM, Ferretti JJ, Karasawa T, Suvorov AN, Lin S, Qin B, et al. Genome sequence of a nephritogenic and highly transformable M49 strain of Streptococcus pyogenes. J Bacteriol. 2008 Dec;190(23):7773-85.

7. Simon D, Ferretti JJ. Electrotransformation of Streptococcus pyogenes with plasmid and linear DNA. FEMS Microbiol Lett. 1991 Aug 1;66(2):219-24.

8. Mashburn-Warren L, Morrison DA, Federle MJ. A novel double-tryptophan peptide pheromone controls competence in Streptococcus spp. via an Rgg regulator. Mol Microbiol. 2010 Nov;78(3):589-606.

9. Ajdic D, McShan WM, McLaughlin RE, Savic G, Chang J, Carson MB, et al. Genome sequence of Streptococcus mutans UA159, a cariogenic dental pathogen. Proc Natl Acad Sci U S A. 2002 Oct 29;99(22):14434-9.

10. Husmann LK, Scott JR, Lindahl G, Stenberg L. Expression of the Arp protein, a member of the M protein family, is not sufficient to inhibit phagocytosis of Streptococcus pyogenes. Infect Immun. 1995 Jan;63(1):345-8.

11. Le Breton Y, Mistry P, Valdes KM, Quigley J, Kumar N, Tettelin H, et al. Genome-wide identification of genes required for fitness of group A Streptococcus in human blood. Infect Immun. 2013 Mar;81(3):862-75.

12. Mashburn-Warren L, Morrison DA, Federle MJ. The cryptic competence pathway in Streptococcus pyogenes is controlled by a peptide pheromone. J Bacteriol. 2012 Sep;194(17):4589-600.
