# Supplementary material for: PptAB Exports Rgg Quorum-Sensing Peptides in Streptococcus
Source: PLoS One. 2016 Dec 19;11(12):e0168461. doi: 10.1371/journal.pone.0168461 (PMC5167397; doi:10.1371/journal.pone.0168461)
Supplement: S1 Table — (DOCX) [file pone.0168461.s005.docx]

**S1 Table. Strains and plasmids used in this study**

| **Strain/plasmid** | **Description** | **Supplemental Reference** |
| --- | --- | --- |
| *S. pyogenes* strains |  |  |
| BNL170 | NZ131 *shp2*_GGG_*shp3*_GGG_; unmarked | (1) |
| BNL193 | NZ131 *shp2*_GGG_*shp3*_GGG_ Δ*rgg3*::*cat*; Cm^R^ | (2) |
| BNL204 | BNL193 with P*_shp3_*-*lux* reporter (pJC219); Cm^R^ Erm^R^ | This study |
| JCC131 | NZ131 Δ*rgg3*::*cat*; Cm^R^ | (3) |
| JCC198 | JCC131 with *luxAB* inserted downstream of *shp3*; Cm^R^ | (4) |
| JCC233 | JCC131 with *gus* inserted downstream of *shp3*; Cm^R^ | (4) |
| JCC208 | NZ131 Δ*pptAB*::*aphA3*; Km^R^ | This study |
| JCC209 | NZ131 Δ*rgg3*::*cat* Δ*pptAB*::*aphA3*; Cm^R^ Km^R^ | This study |
| JCC218 | NZ131 *shp2*_GGG_*shp3*_GGG_ Δ*pptAB*::*aphA3*; Km^R^ | This study |
| NZ131Δ*comR*::*cat* | NZ131Δ*comR*::*cat*; Cm^R^ | This study |
| MGAS8232 | Wild-type M18 *S. pyogenes* isolate; M1 type *comS* allele | (5) |
| MW361 | NZ131 with allelic *comR*_MGAS8232_ replacement; unmarked | This study |
| NZ131 | Wild-type M49 *S. pyogenes* isolate; M1 type *comS* allele | (6-7) |
|  |  |  |
| *S. mutans* strains |  |  |
| JCC263 | UA159 Δ*pptAB*::*aphA3*; Km^R^ | This study |
| MW02 | UA159 Δ*comR*::*spec*; Spec^R^ | (8) |
| MW05 | UA159 Δ*comS*::*spec*; Spec^R^ | (8) |
| MW17 | MW05 with pWAR304; Spec^R^ Erm^R^ | (8) |
| UA159 | Wild-type *S. mutans* | (9) |
|  |  |  |
| Plasmids |  |  |
| pJC219 | 384 bp DNA fragment containing the NZ131 *shp3* promoter fused to *luxAB* and cloned into p7INT; Erm^R^ | (2) |
| pJC250 | 4094 bp DNA fragment containing the NZ131 *pptAB* region cloned into pFED760; Erm^R^ | This study |
| pJC251-kan | pJC250 but with *pptAB* replaced by a kanamycin resistance cassette; Erm^R^; Km^R^ | This study |
| pJC252 | 1989 bp DNA fragment encompassing *pptAB* cloned into pLZ12-Sp; Spec^R^ | This study |
| pJC297 | 3761 bp bp DNA fragment containing the UA159 *pptAB* region cloned into pFED760; Erm^R^ | This study |
| pJC298 | pJC297 but with *pptAB* replaced by a kanamycin resistance cassette Erm^R^; Km^R^ | This study |
| pJC303 | 125 bp fragment containing the *recA* promoter in pLZ12-Sp; Spec^R^ | This study |
| pJC350 | 120 bp DNA fragment containing NZ131 *shp2* cloned behind P*_recA_* in pJC303; Spec^R^ | This study |
| pJC352 | 194 bp DNA fragment containing NZ131 *shp3* cloned behind P*_recA_* in pJC303; Spec^R^ | This study |
| pJC354 | 159 bp DNA fragment containing NZ131 *comS* cloned behind P*_recA_* in pJC303; Spec^R^ | This study |
| pJC371 | 155 bp DNA fragment containing UA159 *comS* cloned behind P*_recA_* in pJC303; Spec^R^ | This study |
| pLZ12-Sp | Shuttle vector encoding spectinomycin resistance; pWV01 origin, Spec^R^ | (10) |
| pOsKar | Source of *aphA3* kanamycin resistance cassette | (11) |
| pWAR195 | *comR* knockout vector | (12) |
| pWAR200 | NZ131 P*_sigX_*-*luxAB* multi-copy reporter; Erm^R^ | (12) |
| pWAR289 | MGAS8232 *comR* region in pFED760; Erm^R^ | This study |
| pWAR304 | UA159 P*_sigX_*-*luxAB* multi-copy reporter; Erm^R^ | (8) |
| pFED760 | pGh9delISS1; temp^S^; Erm^R^ | (8) |

R = resistant; S = sensitive
