# Supplementary material for: PptAB Exports Rgg Quorum-Sensing Peptides in Streptococcus
Source: PLoS One. 2016 Dec 19;11(12):e0168461. doi: 10.1371/journal.pone.0168461 (PMC5167397; doi:10.1371/journal.pone.0168461)
Supplement: S2 Table — (DOCX) [file pone.0168461.s006.docx]

**S2 Table. Primers used in this study**

| **Primer** | **Sequence** | **Description** |
| --- | --- | --- |
| JC175 | CATG**AGATCT**TTAAAATTTCATCCTCCTACG | AS; *shp3* for pJC352 |
| JC316 | GCATG**GCGGCCGC**CAAAATCGTATCCGAACAG | S; *pptAB* region for pJC250 |
| JC317 | GCATG**GCGGCCGC**CCATCCCTGTGATAAATCC | AS; *pptAB* region for pJC250 |
| JC318 | GCATG**TTAATTAA**GAAGATCATTGACTAAGTAGC | S; to delete *pptAB* in pJC250 by inverse PCR |
| JC319 | GCATG**TTAATTAA**GTTTAACATATAATTTCTTTCTAG | AS; to delete *pptAB* in pJC250 by inverse PCR |
| JC320 | CATG**TTAATTAA**CGATACTATGTTATACGC | S; *aphA3* cassette from pOsKar |
| JC321 | CATG**TTAATTAA**AGCGAACTTTTAGAAAAG | AS; *aphA3* cassette from pOsKar |
| JC322 | CATG**GAATTC**ACCTGCTCATCTTCGTAGAC | S; *pptAB* for pJC252 |
| JC323 | CATG**GGATCC**TTGTTCCGTCGTTGTCAC | AS; *pptAB* for pJC252 |
| JC403 | CATG**GCGGCCGC**AATTACCAACAACCTGCTGC | S; *pptAB* region for pJC297 |
| JC404 | CATG**GCGGCCGC**CTGTAATAAAACGACCCTTGC | AS; *pptAB* region for pJC297 |
| JC405 | CATG**AGATCT**GCTGTTTAAAACAAGTAAAAT | S; for inverse PCR on pJC297 |
| JC406 | CATG**TTAATTAA**TTCTTTCTAACTTTGATATACTA | AS; for inverse PCR on pJC297 |
| JC407 | CATG**AGATCT**CTAAAACAATTCATCCAGTAAAATATAA | AS; *aphA3* cassette from pOsKar |
| JC426 | CATG**GCGGCCGC**ATGTTAAAAAAGTATAAGTACTATT | S; *comS*_M1_GAS_ for pJC354 |
| JC427 | CATG**GCGGCCGC**ATGAAGAAAATTTCAAAATT | S; *shp3* for pJC352 |
| JC495 | CATG**GCGGCCGC**ATGAAAAAAGTTAATAAAGC | S; *shp2* for pJC350 |
| JC498 | CATG**AGATCT**TCTAAAATTCTAGCCATAGG | AS; *comS*_M1_GAS_ for pJC354 |
| JC511 | CATG**GCGGCCGC**ATGTTTTCAATTTTAACAAGTATTTTGATG | S; *comS*_UA159_ for pJC371 |
| JC512 | CATG**AGATCT**TATGTCTATTGAATTTGCTTCTC | AS; *comS*_UA159_ for pJC371 |
| LMW30 | GCGTG**GCGGCCGC**AATGACCATCATGATGGGTCGTACCCA | S; *comR* region |
| LMW33 | GCGTG**CTCGAG**TAAAATTTTCTGATCAATGT | AS; *comR* region |
| SHP2-C9-rev-BglII | CATG**AGATCT**ACAAACTAAATATAAGGGTTTCC | AS; *shp2* for pJC350 |
|  |  |  |

S = sense; AS = antisense; boldface indicates restriction site
